# Differences in initial abundances reveal divergent dynamic structures in Gause's predator–prey experiments

**DOI:** 10.1002/ece3.9638

**Published:** 2022-12-18

**Authors:** Lina Kaya Mühlbauer, William Stanley Harpole, Adam Thomas Clark

**Affiliations:** ^1^ Institute of Biology University of Graz Graz Austria; ^2^ Department of Physiological Diversity Helmholtz Centre for Environmental Research (UFZ) Leipzig Germany; ^3^ German Centre for Integrative Biodiversity Research (iDiv) Halle‐Jena‐Leipzig Leipzig Germany; ^4^ Institute of Biology Martin Luther University Halle Germany

**Keywords:** chaos, empirical dynamic modeling, initial abundance, microcosm experiments, nonlinear dynamics, time series analysis

## Abstract

Improved understanding of complex dynamics has revealed insights across many facets of ecology, and has enabled improved forecasts and management of future ecosystem states. However, an enduring challenge in forecasting complex dynamics remains the differentiation between complexity and stochasticity, that is, to determine whether declines in predictability are caused by stochasticity, nonlinearity, or chaos. Here, we show how to quantify the relative contributions of these factors to prediction error using Georgii Gause's iconic predator–prey microcosm experiments, which, critically, include experimental replicates that differ from one another only in initial abundances. We show that these differences in initial abundances interact with stochasticity, nonlinearity, and chaos in unique ways, allowing us to identify the impacts of these factors on prediction error. Our results suggest that jointly analyzing replicate time series across multiple, distinct starting points may be necessary for understanding and predicting the wide range of potential dynamic types in complex ecological systems.

## Graphical abstract

In this manuscript, we show that differences in the initial abundances in a two‐species predator–prey microcosm experiment leads to divergence in their underlying dynamics, which impedes precise forecasting across replicates. We then access the relative contributions of nonlinearity, chaos, and stochasticity to this decrease in predictability. To do this, we analyze time‐series data of two predator–prey systems, produced by Georgii F. Gause in 1936. In these experiments, replicates only differ in their initial abundances.

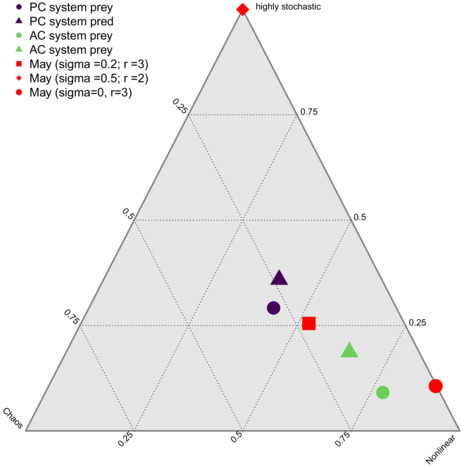

## 1 INTRODUCTION

Accelerating global changes are impacting the global climate and imperiling biodiversity, leading to an increased need for ecology to serve as a predictive science with which to forecast and better manage future ecosystem states (Dietze et al., 2018). A better understanding of complex, and even chaotic dynamics, along with major improvements in methodology, have revealed insights across many systems, and have helped address canonical quandaries such as the ‘Paradox of the Plankton’ (Huisman & Weissing, 1999; Hutchinson, 1961), relationships between biodiversity and stability (Ushio et al., 2018), and the role of stochasticity in structuring real‐world ecosystems (Shoemaker et al., 2020). Nevertheless, further improvements in ecological theory and methods are required to extend these piecewise successes into a broader and more general toolset, with which we can make better forecasts and extend predictive knowledge of why and where forecasts succeed or fail (Pennekamp et al., 2019). A major enduring challenge in analyzing complex dynamics remains the differentiation between stochasticity and complexity in dynamical structures. While stochasticity and complexity can occur simultaneously, and both cause declines in predictive ability, each also leaves behind unique signals, and their effects must be addressed using different methods.

Stochasticity is a process that describes potential intrinsic randomness in the behavior of some real‐world systems. Stochasticity can arise either due to process noise – for example, demographic and environmental variability, which can serve a structuring role in population dynamics – or due to observation error – that is, variability caused by imperfect measurements that do not reflect actual changes in the true system state (Shoemaker et al., 2020). These effects are literally impossible to predict at the level of individual observations but, taken on aggregate across many observations, follow a discernible probabilistic structure.

In contrast to stochasticity, complexity describes processes that are at least in theory perfectly predictable but are nevertheless challenging to predict due to their dynamical structure. Nonlinear dynamics, where a change in the input does not change the output in direct proportion, are very common in natural systems, especially in fast‐reproducing species (Clark & Luis, 2020). A common example is the oscillatory behavior of predator–prey systems (Blasius et al., 2020). These are known to be predictable with simple models as first described by Lotka and Volterra (Lotka, 1925; Volterra, 1926) and experimentally underlined by the work of Gause (1934). However, in more complex systems (e.g., where interaction strengths vary across time or context), models can become substantially more parameter rich, and harder to fit to real data (Karakoç et al., 2020), because differences in predicted abundances can arise due to many different assembly processes, even within a single system (Vellend, 2016).

At its most extreme, minor errors in model form or in estimates of initial system states can lead to prediction errors that grow exponentially over time – a hallmark of chaos, a special case of complexity. Within our definition, chaos is always also nonlinear, as the output is not proportional to the input. After chaos was introduced into ecological studies (May, 1975), it was suggested that if single‐species systems that exhibited chaotic behavior could be found, then it could be expected that a majority of higher diversity ecosystems should behave similarly (Schaffer & Kot, 1985). Inspired by this suggestion, several high‐profile papers identified evidence for chaos in empirical systems and suggested that chaos could play a role in important ecosystem processes, such as the maintenance of diversity (Huisman & Weissing, 1999; Tilman & Wedin, 1991). Similarly, several systems were shown to act chaotically under at least some predefined manipulations of experimental conditions (Costantino et al., 1997; Becks et al., 2005; Graham et al., 2007). Later on, more general worries emerged concerning the interpretation of chaos in field systems, where the sensitivity to initial conditions could be rendered largely irrelevant given sufficiently strong effects of stochasticity (Ellner and Turchin 2005). Despite these potential limitations, empirical studies still regularly find results that suggest that chaos plays an important role in maintaining diversity, at least in some ecological systems (Benincà et al., 2015).

In addition to complexity and stochasticity, declines in the system predictability can also occur due to errors in model specification. Depending on the model chosen, and how it is parameterized, model predictions can vary enormously, even when applied to the same dataset and hypotheses (Wagenmakers et al., 2022). While a wide range of nonparametric and model‐free analytical approaches have been developed, model‐dependent analysis remains one of the most common approaches for investigating ecological dynamics (Dietze, 2017). These effects of model choice can be especially problematic for attempts to separate the effects of stochasticity versus chaos. For example, Lyapunov exponents, which are the main metric used to examine chaotic behavior in empirical systems, are still mainly estimated by fitting models to data, meaning that errors in model choice will lead to incorrect concussions about the structure of underlying dynamics. However, as early as 1990, Sugihara and May suggested methods for avoiding this potential bias by estimating Lyapunov exponents using a more flexible nonparametric method, which we will use in this study to identify chaotic behavior in the investigated systems.

Here, we present an approach for quantifying the influences of stochasticity, nonlinearity, and chaos on ecological dynamics. We apply this approach to study the potential effects of these three processes on the predictability of a predator–prey system, using a set of Gause's predator–prey microcosm experiments (Gause et al., 1936) as case study.

As deterministic chaos is commonly defined as bounded but unpredictable fluctuations of state variables that are sensitive to initial conditions (Eckmann & Ruelle, 1985), we chose a set of experiments in two different predator–prey systems, each of which includes multiple replicates that differ only in their initial abundances. Microcosm predator–prey systems are well‐suited for the problem, as they exhibit complex dynamics due to oscillations but are nevertheless highly controlled in their environmental conditions. Importantly, because we can assume relatively low observation error in Gause's simple microcosm experiments due to their small size and frequent measurements, we can therefore attribute prediction errors between replicates to complexity (chaos or nonlinearity), deterministic variation (e.g., oscillations), and remaining variability (process noise). We reanalyze these datasets using the non‐parametric approach (empirical dynamic modeling) (Ye et al., 2015) proposed by Sugihara and May (1990) to compare their cross‐prediction abilities (i.e., predictions of dynamics in one replicate based on observations in other replicates).

Stochasticity, nonlinearity, and chaos will all cause prediction ability to decrease with increasing distance in initial abundances between replicates, but each leaves a unique signal. If differences are due to nonlinear dynamics, then we expect that local linear approximations of the system should provide poor predictions of dynamics (Sugihara & May, 1990; Ye & Sugihara, 2016). To examine the effects of chaotic dynamics, we determine the corresponding Lyapunov exponents of the cross‐predictions. If differences among replicates are driven by chaos, we would expect to find that cross‐predictions decline quickly as a function of differences in initial abundances between replicates and, more importantly, that the Lyapunov exponents for the system are positive. Finally, if differences in cross‐predictions are driven by stochasticity, then the deterministic component of dynamics, and corresponding estimates of time‐varying species interaction coefficients, should on average, be consistent across experimental replicates. We are treating stochasticity as the remaining variability, the prediction error that results not from chaos, nonlinearity, deterministic variation (e.g., oscillations), or observation error.

Jointly, these unique signals should therefore allow us to estimate the relative impacts of nonlinearity, chaos and stochasticity on predictability in our study system.

## 2 MATERIAL AND METHODS

### 2.1 Data

The experimental microcosm data was digitized as part of a previous study (Mühlbauer et al., 2020) from Gause et al. (1936). To analyze the effects of different initial conditions on the dynamics of Gause's predator–prey microcosm experiments, we chose two different predator–prey datasets, each of which included multiple experimental replicates that differed only in the initial abundances of the predator and prey species (Gause et al., 1936). The first dataset consists of 19 experimental replicates, showing the interaction of the predator *Paramecium bursaria* and prey species *Saccharomyces exiguus* (PS‐system). The other dataset shows interactions between the predator *Cheyletus eruditus* and prey species *Aleuroglyphus agilis*, with semolina flour as a feedstock (AC‐system). Whereas the PS‐system dataset only contains experimental replicates with unique initial abundances, in the AC‐system two replicates were conducted using identical initial abundances. The abundances were reported as the number of individuals (AC‐system) and as individuals per 1/10 mm^3^ (*S. exiguus*) and per 0.5 cm^3^ (*P. bursaria*) in the PS‐system. The raw data of Gause's predator–prey experiments were interpolated using a cubic spline to infer missing data points and to generate the necessary temporal density of data points for the EDM fitting. Although time‐series length is an important limitation in time‐series analyses, temporal density is more important for empirical dynamic modeling (Munch et al., 2020). Time steps were chosen based on the length of the time series – 0.5 days for the shorter *Paramecium bursaria/Saccharomyces exiguus* series and 1 day for the *Aleuroglyphus agilis/Cheyletus eruditus* data.

### 2.2 Empirical dynamic modeling

All analyses were conducted using the R programming language (R Core Team 2019) in version 3.6.2. All EDM analyses were conducted using the R package rEDM version 0.7.4 (https://github.com/ha0ye/rEDM) based on Sugihara (1994). To calculate the Lyapunov exponents and the corresponding mean absolute error (MAE), forecasts predicting dynamics 1 to 10 timesteps into the future were used. Time delay size was set to 1 timestep for all analyses, leading to time intervals of 1 day (AC‐systems) or 1/2 day (PS‐system), which maximized cross‐validated goodness‐of‐fit. We used the simplex() function of the R package rEDM, which uses the projection nearest neighbor forecasting method, to determine the best embedding dimension (E) (Sugihara & May, 1990). Following ‘best practices’ for EDM, E that minimizes the cross‐validated MAE from observed to predicted values was used (i.e., we chose E to maximize cross‐validated predictive ability).

**FIGURE 1:**
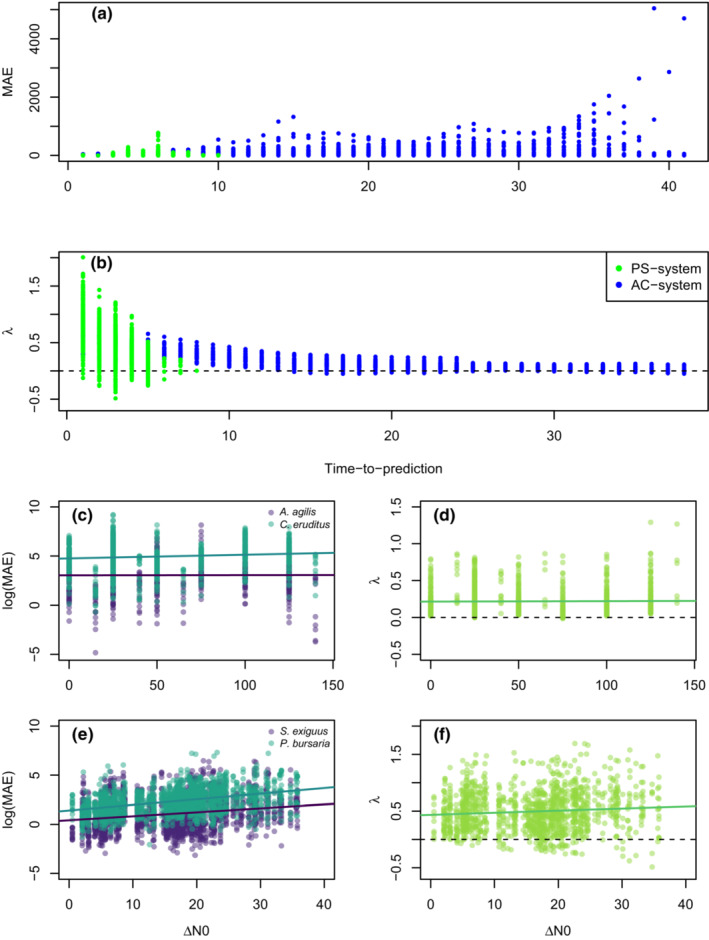
Univariate effects of prediction intervals (time‐to‐prediction) and distance in initial abundances (Δ*N*0) on prediction quality (mean absolute *error (MAE)*) and Lyapunov exponents (λ) in pairwise cross‐predictions in multivariate (a, b, d, and f) and single variate embeddings (c and e). (a, b) show the effect of the prediction length on MAE and Lyapunov exponent in the predator–prey system of *Aleuroglyphus agilis* (prey) and *Cheyletus eruditus* (predator), with wheat as a feedstock (light green) and *Paramecium bursaria* and *Saccharomyces exiguus* (blue). (c, e) show the effect of differences in initial abundances on MAE for both species in each system. (d, f) represents the relationship between Lyapunov exponent and Δ*N*0. (c) *A. agilis*: *R* ^2^ = −.0009; *p* = .87; b = 0.0002 *C. eruditus*: *R* ^2^ = .01; *p* < .001; b = 0.004 d. *R* ^2^ = −.0008; *p* = .68; b = 0 e. e. *S. exiguus*: *R* ^2^ = .04; *p* < .001; b = 0.04 *P. bursaria*: *R* ^2^ = .04; *p* < .001; b = 0.04 f. *R* ^2^ = −.008; *p* < .004; b = 0.

To identify the S‐Map neighbor localization exponent (θ), the s‐map() function was used (Sugihara, 1994). This function is similar to classic simplex projection, but makes predictions based on a series of locally weighted linear regressions rather than locally weighted means. Again, following best practices for EDM, θ that minimizes cross‐validated MAE was chosen. Because θ describes the strength of the local weighting function, the best fitting θ tends to be higher in systems with nonlinear dynamics (i.e., because local linear approximations perform poorly when applied to more distant system states) (Sugihara, 1994). Note that in the rEDM package, model performance is automatically tested for out‐of‐sample fit using leave‐one‐out cross‐validation, which provides strong protection against over‐fitting models, even when timeseries are very short. Single variable embeddings were fitted to each of the single species time series using the s_map() function and the previously determined parameters. This method was used for Figure 1 to emphasize the differences in predicting predator and prey abundances. The time series of predator and prey were also fitted simultaneously with the s‐map algorithm, using block_lnlp() which allows multivariate time‐series data to be used as different dimensions for reconstructing the attractor. Single and multivariate approaches are, in theory, equivalent following Taken's Theorem, although there are cases where multivariate embeddings can improve the prediction skill, for example, when observation error is high (Chang et al., 2021; Deyle & Sugihara, 2011; Sauer et al., 1991). In the presented systems, the goodness‐of‐fit only differs slightly between approaches (see Supplement). S‐mapping regression coefficients were extracted from the block_lnlp() function for each time step, which uses the s_map algorithm with multivariate embeddings. These describe the relative weight given to each dimension of the attractor by the local regression algorithm, and can be interpreted as time‐varying interaction coefficients in a multivariate embedding (Deyle et al., 2016; Karakoç et al., 2020).

### 2.3 Prediction of dynamics across replicates

The resulting coefficients of the EDM models were used to perform cross‐predictions between experimental replicates differing in their initial abundances. These cross‐predictions were conducted with the s_map() function, using the time series from the one replicate to train the algorithm, and then applying the trained algorithm to predict dynamics for a second replicate, and vice versa. For each of these comparisons, we then calculated the resulting mean absolute error (MAE). E was set to 2 for all comparisons, to prevent prediction failures due to differences in E between pairs. Nevertheless, this had minimal effects on results, as goodness‐of‐fit was maximized at E = 2 in all cases except for one experimental replicate in the PS‐system, for which E = 3 provided the best goodness‐of‐fit. Note that E = 2 is also the theoretically expected embedding dimension for a system with two species (Ye et al., 2015).

### 2.4 Chaos

The Lyapunov exponent is a common measure used to quantify divergence over time of nearby points in phase space. The Lyapunov exponent refers to the rate parameter of the equation that describes how this initial distance between nearby points changes over time. If the Lyapunov exponent is positive, the initial separation vector grows, that is, the trajectories diverge exponentially – which is an indication of chaos. In contrast, if the Lyapunov exponent is negative, then trajectories converge over time, which is an indication for a locally stable equilibrium. As the MAE is a measure for the absolute distance between the predicted and observed points, it can therefore act as an estimate of distance in the divergence over time, like that expressed by the Lyapunov exponent. This method was initially proposed by Sugihara (1994). To estimate the Lyapunov exponent, we therefore fitted a linear model of the relationship between log (MAE) and the number of time steps of the time‐to‐prediction interval. We then used the slope of this regression as an estimate of the Lyapunov exponent, λ, following the expression:

(1) $$\epsilon \approx e^{\lambda t}\epsilon_{0}$$

(2) $$\log\left(\epsilon \right)\approx \log\left(e^{\lambda t}\epsilon_{0}\right)$$

(3) $$\log\left(\epsilon \right)\approx \log\left(e^{\lambda t}\right)+\log\left(\epsilon_{0}\right)$$

(4) $$\log\left(\epsilon \right)\approx \lambda t+\log\left(\epsilon_{0}\right)$$

As a significance measure for the Lyapunov exponent, the p‐value for the slope of the linear model of log (MAE) vs. time was used.

### 2.5 Difference in initial distance

To test whether these difference in prediction ability resulted from different starting positions in phase space, we used the Euclidean distance as a metric for initial distance between replicates:

(5) $$\Delta N0=\sqrt{\left({\left(N_{\text{predator},1}-N_{\text{predator},2}\right)}^{2}+{\left(N_{\text{prey},1}-N_{\text{prey},2}\right)}^{2}\right)}$$

where *N*_prey,1_ and *N*_predator,1_ are, respectively, the initial abundance of the prey and predator species in experimental replicate 1, and *N*_prey,2_ and *N*_predator,2_ are the initial abundance of the prey and predator species in experimental replicate 2. Larger Δ*N*0 values therefore indicate a greater divergence in initial predator and/or prey abundances between experimental replicates. Comparisons among metrics were conducted using ordinary least squares regression, reported in terms of *p*‐value, effect size, and *R*‐squared (see figure legends for specific values).

### 2.6 Estimating contributions of stochasticity, nonlinearity, and chaos to prediction error

To assess the relative contributions of nonlinearity, chaos, and stochasticity on reductions in prediction ability, we estimated the associated fraction of the mean square error (MSE) attributable to each of these three components.

First, we quantify total prediction error in the timeseries, less the effects of linear deterministic variability, by calculating the MSE of the EDM fit with θ = 0 (hereafter ‘total variation’). Note that θ = 0 implies a perfectly linear model, that is, that dynamics around all points in phase space can be described using a single linear function of the current state and corresponding time lags. To quantify effects of nonlinearity, we subtract the MSE of the EDM fit with ‘best’ θ, that is, the value which minimizes MSE, from the total prediction error (hereafter $\mathrm{MS}E_{\text{bestθ}}$). The difference between these two quantities thus represents the net effect of deterministic nonlinear dynamics on predictive ability.

Second, to quantify effects of chaotic dynamics on predictive ability, we calculate an estimate of Lyapunov Exponent ($\lambda_{\mathrm{est}})$ by comparing the root mean square error (RSME) between each time step *i* ($\mathrm{RMS}E_{t_{i}})\;$and time step *i* + 1 $\left(\mathrm{RMS}E_{t_{i+1}}\right)\;$of the EDM fit with the ‘best’ θ using Equation (6a‐b). Note that we write this in term of RMSE, as this is a measure of distance between observations and predictions: as discussed in Sugihara and May (1990), this metric can be used as a rough indicator of divergence rate (i.e., treating the observation as one replicate, and the EDM‐based prediction as a second replicate with starting state near the observed replicate), and is thus a direct and efficient metric for measuring the Lyapunov Exponent. We use RMSE in this calculation rather than MSE or MAE because RMSE can be related directly to total variance, which allows us to compare the magnitude of its effect to that of other sources of variation. However, for the range of values that we compare here, RMSE is very similar to MAE or Euclidian distance (i.e., both have almost identical magnitudes), meaning that RSME corresponds closely to average distance between predictions and true values. Specifically, we calculated the Lyapunov Exponent as the exponential rate at which RSME grows with prediction time horizon, as:

(6a) $$\mathrm{RMS}E_{t_{i+1}}=\mathrm{RMS}E_{t_{i}}\exp\left(\lambda_{\mathrm{est}}\;\left(t_{i+1}-t_{i}\right)\right)$$

(6b) $$\lambda_{\mathrm{est}}=\log\left(\mathrm{RMS}E_{t_{i}}/\mathrm{RMS}E_{t_{i+1}}\right)$$

For $\lambda_{\mathrm{est}}>0$, the quantity $\mathrm{RMS}E_{0}\exp\left(\lambda_{\mathrm{est}}\right)$ thus represents the total amount of divergence between predictions and true dynamics expected per timestep due to chaotic dynamics, where $\mathrm{RMS}E_{0}$ is the total prediction error driven by mechanisms other than chaos. This implies that

(7a) $$\mathrm{RMS}E_{\text{bestθ}}=\mathrm{RMS}E_{0}\exp\left(\lambda_{\mathrm{est}}\right)$$

(7b) $$\mathrm{RMS}E_{0}=\mathrm{RMS}E_{\text{bestθ}}/\exp\left(\lambda_{\mathrm{est}}\right)$$

We therefore calculate the prediction error due to chaotic dynamics following Equation (7c) as

(7c) $$\mathrm{MS}E_{\text{chaos}}=\left(\mathrm{MS}E_{\text{bestθ}}-\text{RSMS}{E_{0}}^{2}\right)=\mathrm{MS}E_{\text{bestθ}}\left(1-\exp\left(-\lambda_{\mathrm{est}}\right)\right)$$

Finally, we assume that all remaining prediction error not due to nonlinear or chaotic dynamics is due to stochasticity. To calculate relative contributions of each of these components to total prediction error, we divide each quantity by total error. To assure that the chaos fraction stays below 1, we assume an RSME resulting from chaos is 0, if $\lambda_{\mathrm{est}}$ < 0. Code for implementing and plotting these analyses can be found in the Supplement S2.

## 3 RESULTS

The overall goodness‐of‐fit in all experiments in multivariate embeddings is E_2_ = 0.984 (where E_2_ is the coefficient of efficiency, which is similar to *R* ^2^, but measures scatter around the 1–1 line rather than around a fitted regression line) for the PS‐system, while the fit for the single variate embeddings is slightly lower for the prey (E_2_ = 0.983) than for the predator (E_2_ = 0.99). In the AC‐system, the fit of the multivariate embeddings is slightly higher (E_2_ = 0.988). In the single variate embeddings, the fit is also better for the predator (E_2_ = 0.998) than for the prey (E_2_ = 0.987). Note that for relatively long timeseries, these very high levels of predictive ability are common for EDM (Perretti et al., 2013).

The MAE of cross‐predictions increases significantly with increasing distance of initial abundances (Δ*N*0) in both species in the PS‐system, while the relationship between Lyapunov exponent and Δ*N*0 is less clear (Figure 1c,e). However, in the AC‐system, MAE first increases and then decreases again, and there is less indication that the cross‐prediction ability decreases with the distance in initial abundances. Especially striking is that the Lyapunov exponents only exhibit positive values, one hallmark of at least partly chaotic dynamics (Figure 1e).

The influence of the length of cross‐prediction shows an interesting pattern of increasing and decreasing MAE, with the highest MAE values in time steps beyond 35 for the AC‐system. Note that the length of the time series differs between PS‐system (up to 7 days) and AC‐system (up to 44 days). After eight time steps in the PS‐system, the MAE drops and stays relatively low (Figure 1a), which is potentially indicative of survival bias – that is, because replicates with less complex dynamics also tended to persist for longer. The MAE in the AC‐system increases with each time step, because a cross‐prediction is possible up to 41 time steps for most replicates. The effect of time on Lyapunov exponents underpins this finding, as it decreases quickly (PS‐system), while the AC system exhibits positive values near zero over the whole‐time range (Figure 1b).

**FIGURE 2:**
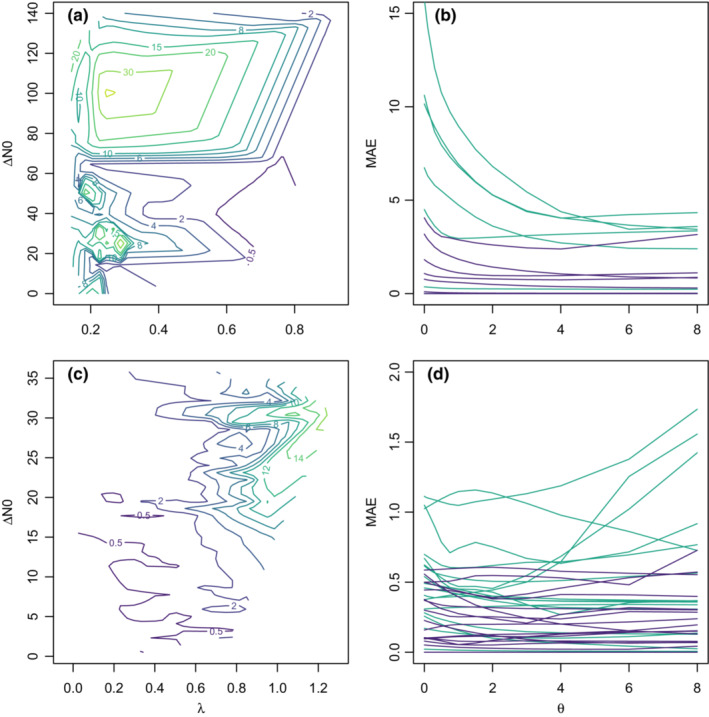
Interactions between Lyapunov exponent (λ), distance in initial abundances (Δ*N*0) and nonlinearity (*θ*) influencing the prediction ability (mean absolute *error (MAE)*) in pairwise cross‐predictions of experimental replicates in the predator–prey‐system of *Aleuroglyphus agilis* (prey) and *Cheyletus eruditus* (predator), with wheat as a feedstock (a, b) and *Paramecium bursaria* and *Saccharomyces exiguus* (b, c). A, c. colored contour lines show standardized MAE. Violet parts show an MAE < standard deviation/2, which corresponds to a rough rule of thumb for identifying “good” model performance (e.g., Moriasi et al., 2007). Only significant (*p* < .05) Lyapunov exponents are included. Self‐predictions are excluded. Lyapunov exponents and MAE are represented as means over all time steps. (b, d) Each line represents the MAE, in relation to *θ*, of one experimental replicate, violet represents the predator species, green the prey species.

In the AC‐systems, high MAE values occur over the whole range of Δ*N*0, although MAE decreased somewhat when both Δ*N*0 and Lyapunov Exponents were low (Figure 2a). Thus, prediction errors are related to both chaos, and to differences in dynamic form that vary as a function of starting abundance. In contrast, in the PS‐system, high MAE values only appear in medium high or high Δ*N*0 and high Lyapunov Exponents. Again, these dynamics are thus associated with both chaos and differences in starting points (Figure 2c). Surprisingly, only the AC‐system shows nonlinear behavior, that is, with forecast skill increasing for θ > 0 (Figure 2b), results are mixed for the PS‐system (Figure 2d).

**FIGURE 3:**
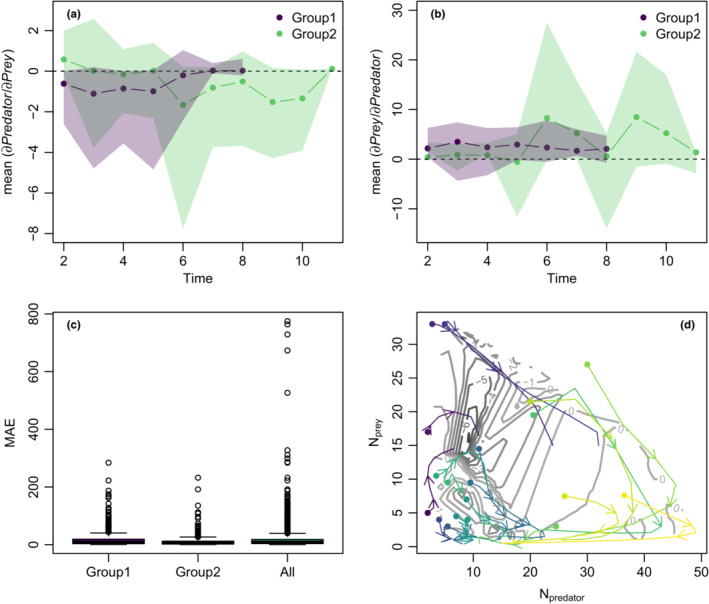
Indications for two different kinds of dynamics in the predator–prey system with *Paramecium bursaria* and *Saccharomyces exiguus*. Experimental replicates were grouped by their starting point in phase space (see points in d.). (a, b) The mean effect of predators on prey and vice versa over time is compared between groups. Shaded areas show the confidence intervals (standard error from the mean effect of predator on prey and vice versa). (c) The total mean absolute *error (MAE)* in comparison between groups and with cross‐predictions between groups (all), self‐predictions were removed. (d) Relationship of effect of predators on prey (δPredator/δPrey) and observed time series in phase space. Gray contour lines show the strength and direction of the interaction. Colored arrows show observed predator and prey dynamics for all experimental replicates.

To further investigate how dynamic form varied as a function of position in phase space in the PS‐system, we extracted time‐varying species interaction coefficients from s‐maps fitted with the optimal θ. These results indicated major differences in interactions across system states. Replicates appear to fall into two regions, showing either exceptionally negative or positive effects of predators on prey. There also seems to be a region of phase space that the observed time series cannot cross, although the initial abundances are evenly distributed over the phase space (Figure 3d). Separating the experimental replicates into two groups, one with strong negative interactions and one with strong positive interactions, divided by the uncrossed region in phase space, reveals that the highest MAE values occur in cross‐predictions that compare dynamics between these two groups (Figure 3c). In particular, the mean effect of prey on predators in group 1 vary around 0, while they stay positive in in group 2 (Figure 3a). Additionally, the effect of predator on prey in group 1 starts as negative, but approaches zero after 8 days as both species died out. In group 2, in contrast, the effect of predators on prey starts as positive, but then decreases and approaches zero only at day 11 (Figure 3a). In brief, the two groups show different interaction patterns of predators and prey in time and space, which result in a lower predictability between groups.

**FIGURE 4:**
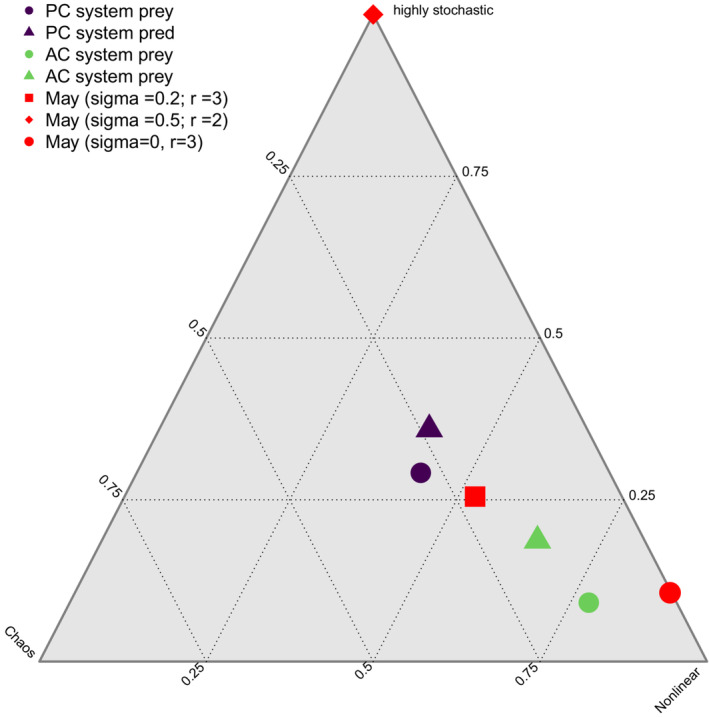
Estimated fractions of chaos, nonlinearity, and stochasticity on the prediction error (rsme) in the analyzed microcosm experiments (violet, green) and three variations of a chaotic model (May, 1975) with different amounts of noise (sigma) and different growth rates (r) in red.

Taken together, these results suggest that stochasticity, nonlinearity, and chaos all jointly contribute to prediction error in both the AC‐ and PS‐systems (Figure 4). The PS‐system shows weaker effects of nonlinearity, and stronger effects of stochasticity, than the AC‐system, whereas the AC‐system shows weaker effects of chaos. As a ‘sanity check’ for the comparison, we also plotted the position of a simulated time series following May's chaotic discrete‐time logistic growth model (May, 1975). As expected by theory, our approach accurately predicts that higher growth rates in the model lead to more chaotic dynamics, and that stronger stochastic forcing (driven by random variation added to initial abundance dynamics) lead to stronger signals of stochasticity (Figure 4, red).

## 4 DISCUSSION

Our findings show that even in Gause's relatively simple and controlled microcosm experimental systems, chaos, nonlinearity, and stochasticity each contribute to decreases in prediction ability. Note that cross‐prediction skill, that is, the ability to predict dynamics in one replicate based on model fitted to another replicate, does not correlate solely with the difference in initial abundances Δ*N*0. In particular, we find that in many cases, replicates diverged quickly even when they started with quite similar initial abundances (as indicated by positive Lyapunov exponents), potentially indicating that divergence in system dynamics is driven more strongly by chaos than by the position in phase space of the replicates, as we discuss below.

### 4.1 Chaos

There are indications of chaotic dynamics in most experimental replicates and time steps. Although, several other empirical studies have identified chaotic dynamics under predefined conditions (Becks et al., 2005; Costantino et al., 1997; Graham et al., 2007), they typically lack model‐independent derived Lyapunov exponent estimates. Model‐based estimates – that is, those derived by fitting a model to data, and then analyzing the dynamics of the fitted model – are highly influenced by model choice, which has often been criticized (Sugihara, 1994). Moreover, in this study, indications for chaos could be detected for a two‐species system, without manipulating experimental conditions except for the different initial abundances. This finding indicates that dynamics in other low‐dimensional, unmanipulated systems could potentially also experience chaos and divergent dynamical trajectories based solely on small changes in initial abundance, for example, from harvesting as has been shown for fish populations (Anderson et al., 2008). Similar links between chaos and ecosystem management have also been suggested previously by Berryman and Millstein (1989), who proposed that chaos in ecological systems may be disproportionately common in response to human impacts that induce high growth rates or inhibit negative feedback loops.

Our primary evidence for chaotic dynamics is the positive values that we estimate for Lyapunov exponents across replicates – that is, indicating that small differences in initial abundances grow exponentially over time. The AC‐system shows solely positive Lyapunov exponents, whereas the PS‐system shows some negative Lyapunov exponents, but across a relatively wide range of Δ*N*0 values. Interestingly, the Lyapunov exponent converges to zero across all replicates over long‐time intervals. As positive Lyapunov exponents show the divergence of trajectories, and therefore chaotic dynamics, the decrease in Lyapunov exponents could show non‐chaotic behavior. However, this pattern could also be explained by the dependence of estimates on the time‐to‐prediction interval. Even in chaotic systems, species abundances are bounded within a relatively narrow range of potential values (i.e., above zero, and below some carrying capacity), meaning that over time, the average abundances of replicate time series tend to converge (Ellner & Turchin, 1995). Consequently, across wide time intervals and given bounded total population sizes, the Lyapunov exponent must approach zero, and may even become negative, regardless of whether or not dynamics are chaotic. Sadly, this problem is inescapable in most empirical systems, as there must always be at least some bounds on population growth. Despite these empirical limitations, another observation suggests that the dynamics could be partly chaotic, especially in the PS‐system: the observed time series plotted in phase space cross for several predator/prey ratios. In both systems, cases can be found where the time series diverge after they reached the same abundance of predator and prey – that is, they show different dynamics, rather than converging to a single trajectory after crossing. This finding suggests that small differences between the replicates lead to big changes in dynamics – a hallmark of chaos.

### 4.2 Nonlinearity

In the PS system, the existence of two different dynamic regimes, divided by the “uncrossable” region in phase, decreases the cross‐predictability between these groups. Besides the differences in interaction coefficients, group 1 shows mainly nonlinear behavior and the group 2 linear behavior. This nonlinearity is likely due to sedimentation of *S. exiguus*, which occurs in high concentrations, as already noted by Gause et al. (1936). Consequently, the predator is not able to consume the prey, which leads to differences in interaction coefficients between groups. This finding indicates that the connection between chaos and management could occur due to mechanisms that vary as a function of species abundances.

### 4.3 Stochasticity

The fact that we find nonlinear dynamics, but similar interaction coefficients between replicates in the AC‐system, suggests that stochasticity also contributes to the divergences of replicates. Specifically, similar interaction coefficients show that average changes in abundance dynamics can be predicted using roughly the same functional forms and parameter values across replicates. In contrast, the fact that we find relatively high prediction error suggests that there is a substantial scatter in realized abundances that fall around these average estimates. Interestingly, similar indications were also identified by Gause, who reported variability in demographic data for the AC‐system (Gause et al., 1936).

### 4.4 Estimating contributions of stochasticity, nonlinearity, and chaos to prediction error

Taken together, our findings show that there are indeed indications for contributions of chaotic dynamics to the prediction error, as well as effects of nonlinearity and stochasticity. In many cases, our results suggest that chaos is more likely to be detected in systems with strongly stochastic dynamics, potentially indicating an interactive role of these processes in reducing predictive ability. Additionally, it shows that chaos, at least within our definition and approach, is always also nonlinear, as the output is not changing proportionally to the input. This is reflected in the chaotic dynamics in Robert May's model with a growth rate of 3, which are accurately positioned as highly chaotic, but also show nonlinear behavior (Figure 4, red point). Although we cannot provide a clear‐cut partitioning between the effects of stochasticity and chaos, we are able to differentiate between purely stochastic forcing (Figure 4, red diamond), chaotic dynamics (Figure 4, red point) and a combination of both (Figure 4, red square).

These findings underpin the importance of classifying and quantifying stochasticity, chaos, and nonlinearity in systems prior to analyses, in order to properly choose prediction intervals and modeling methods (Figure 4). Also, the presented approach could lead to further insights, by comparing the locations of different systems in Figure 4. By using multiple time series from different real‐world systems, attributes that lead to differences in the amount of stochasticity, nonlinearity, and chaos could be investigated.

Despite the relatively short time series that we consider here, especially for the PS‐system, this study provides a comprehensive investigation of the effects of chaos, nonlinearity, and stochasticity on predictability in these systems. As our results show, the resulting goodness‐of‐fit of the time series can be considered good, despite the short time‐series length (Appendix S1, Figures 1, 2, 3, 4). Although time series length can limit the applicability of EDM, previous simulation studies have shown that even very short timeseries (e.g., just 5 sequential replicates) can be applied to make effective forecasts with EDM, given that data are available from sufficiently many replicates, which is evidentially the case with the Gause data, given the observed goodness of fit (Clark et al., 2015; Hsieh et al., 2008). While the application of ecological theory derived from microcosm experiments to real‐world ecosystems is always difficult, these experiments nevertheless reveal insights in the kinds of population dynamics that are possible under controlled conditions (Currie, 2020). In particular, Crone and Molofsky (1998) argue that microcosm experiments can be informative if demographic structure is not manipulated and the model is at least partly mechanistic. These factors are thus major advantages of the data and methods that we apply here. First, the only difference among the experimental replicates is in their initial abundances, and the replicates are not manipulated in terms of demographic structure or experimental conditions (Gause, 1934). Second, EDM avoids the problems of model dependence when predicting dynamics, which is especially important for extracting Lyapunov exponents as a metric for chaotic dynamics – that is, because our methods are nonparametric, our results do not merely represent a peculiarity of the particular functional form that we chose to use for our model. Lastly, by extracting estimates of interaction coefficients via s‐mapping, we are able to explain our results in terms of interactions between predator and prey species, and thus, the divergent dynamics that we identify can, at least partly, be described in terms of a human‐understandable, mechanistic model (Deyle et al., 2016).

The findings of this study demonstrate the importance of the joint effects of chaos, nonlinearity, and stochasticity on predictability. This work suggests that even in simple microcosm experiments, misleading forecasts can occur due to all three of these processes. Nevertheless, by identifying the root cause of prediction errors, it may be possible to counteract some of these impacts, and identify additional data or methods that could, potentially, improve model predictions. In cases where errors are driven by stochasticity, these errors can be mollified via broader data collection, and by focusing predictions on system averages rather than on individual dynamical trajectories (for which predictions will always be poor). Moreover, even in systems which exhibit large amounts of stochasticity, it will often be possible to extend predictions over longer time intervals, as long as the average effect of underlining stochastic forcing is known and included in the model. In contrast, to overcome nonlinearity as a barrier in ecological forecasting, careful model choice, or the application of flexible non‐parametric methods such as EDM, might help alleviate prediction error and allow accurate predictions. Finally, in chaotic systems, predictions will usually only be accurate over short timeframes, even given good model choice and large amounts of data.

Our results indicate that multiple shorter time series with different starting abundances could perform as well, or even better, than those parameterized with a single longer time series, which is quite encouraging for future studies. This would make some sense, as these multiple short time series are able to more effectively explore the full possible extent of dynamics across state space and thus provide predictions for a wider range of dynamic types. Some similar results have been shown for EDM using spatial replicates (Clark et al., 2015) and species replicates (Hsieh et al., 2008), and in theoretical studies of other models for systems with varying initial abundances (Pascual & Kareiva, 1996). In general, these results suggest that extending the range of conditions across which data are collected may be more important than collecting long‐term data, at least in some systems – which could help greatly increase predictive ability in many ecological systems using currently available data.

## AUTHOR CONTRIBUTIONS

**Lina Kaya Mühlbauer:** Conceptualization (lead); formal analysis (lead); writing – original draft (lead). **William Stanley Harpole:** Supervision (supporting); writing – review and editing (lead). **Adam Thomas Clark:** Supervision (lead); writing – review and editing (lead).

## CONFLICT OF INTEREST

The authors declare no conflict of interest.

### OPEN RESEARCH BADGES

This article has earned Open Data, Open Materials and Preregistered Research Design badges. Data, materials and the preregistered design and analysis plan are available at [insert provided URL(s) on the Open Research Disclosure Form].

## Supporting information


Appendix S1.

## Data Availability

All data used in this manuscript are publicly available in the GauseR package on the CRAN server and at https://doi.org/10.1002/ece3.6926.
