# Supplementary material for: Differences in initial abundances reveal divergent dynamic structures in Gause's predator–prey experiments
Source: Ecol Evol. 2022 Dec 18;12(12):e9638. doi: 10.1002/ece3.9638 (PMC9760897; doi:10.1002/ece3.9638)
Supplement: Supplementary file 1 — Appendix S1. [file ECE3-12-e9638-s001.docx]

**Appendix I**

**Glossary**

**Attractor**

A certain set of values a dynamical system tends to converge to, tracing a multidimensional path across the state space. This concept applies even in highly complex, nonlinear, or chaotic dynamics, which also follow certain equations.

**Embedding dimension**

The dimensionality – and therefore a proxy for the complexity – of the resulting state space of a dynamical system.

**Empirical dynamic Modelling (EDM)**

An approach that uses state space reconstruction to analyze and predict nonlinear dynamics directly from time-series data. The aim of EDM is to reconstruct the attractor of the dynamic system from which the time series is derived. Most basically, this attractor can be reconstructed if all the state variables that produce the attractor are known, and long-term time series data are available for each of them. A potential challenge for EDM is that even in a simple system, not all of the state variables can be observed. However, Taken’s theorem states that unobserved variables can be replaced with time-lagged projections of a single observed variable without any loss of predictive ability (Takens 1981). Thus, projections of the future dynamics of the system can be inferred based on these time-lagged states, allowing EDM to be applied even without full information about a system (Ye and Sugihara 2016).

**Lyapunov exponents**

In dynamic systems theory the Lyapunov exponent is a measure to quantify the divergence over time of two points in state space. Two trajectories in state space have an initial distance that is assumed to grow or shrink with a certain exponential rate. If the Lyapunov exponent is zero, the initial distance does not change. If it is negative, this signals convergence of disparate trajectories, which is usually indicative of a locally stable equilibrium. In contrast, if the Lyapunov exponent is positive, the separation initial distance grows over time. Because the growth rate of this distance is exponential, the system becomes effectively unpredictable over long time periods, since small deviations or prediction errors are rapidly magnified. In other words, small distances in the initial state lead to big changes in the dynamics - one condition for chaos (Munch, Brias, et al. 2020).

**Non-parametric**

Unlike parametric modelling, which typically assumes constant parameters across time, EDM allows parameters to vary over time, and therefore, reduces the dependence of inferences on the chosen dynamical form of a model. Non-parametrical approaches reduce necessary assumptions about underlying equations and parameters to a minimum, because predictions are directly derived from observations.

**Prediction error**

The difference between the observed state of a system and the predicted values derived from the chosen model.

**State space**

A multidimensional space, in which the dimensions represent the state variables of the system. Critically, to effectively reconstruct system dynamics, the state space must be sufficiently high dimensional that it can represent each possible combination of the state variables as a unique point in state space.

Fig. I1: Interpolated values and raw data of Gause's ACs-system experiments (Gause, Smaragdova et al. 1936). *Aleuroglyphus agilis* (prey) and *Cheyletus eruditus* (predator) grown for up to 36 days with semolina flour as a feedstock. Points show reported data points, lines show interpolated values for each day. The abundances are reported as number of individuals.

Fig. I2: Interpolated values and raw data of Gause's PS-system experiments (Gause, Smaragdova et al. 1936). *Paramecium bursaria* (prey) and *Saccharomyces exiguus* (predator) grown for up to 7 days. Points show reported data points, lines show interpolated values for each day. The abundances are reported as individuals per 1/10 mm^3^ (*S. exiguus*) and 0.5 cm^3^ (*P. bursaria*).

Fig. I3: **Fit of self-predictions using multivariate embeddings in EDM** of the predator-prey-system *Saccharomyces exiguus* (prey) and *Paramecium bursaria* (predator) (Gause, Smaragdova & Witt 1936). Dashed line represents one-to-one line, as a goodness-of-fit measure E_2_ is shown, which quantifies the difference of points to the one-to-one line.

Fig. I4: **Fit of self-predictions using EDM**. Left panel shows *Saccharomyces exiguus* (prey) and the right panel shows *Paramecium bursaria* (predator) (Gause, Smaragdova & Witt 1936). Dashed line represents one-to-one line, as a goodness-of-fit measure E_2_ is shown, which quantifies the difference of the points to the one-to-one line.

Fig. I5: **Fit of self-predictions using multivariate embeddings in EDM** in the predator-prey-system *Aleuroglyphus agilis* (prey) and *Cheyletus eruditus* (predator) with wheat as a feedstock (Gause, Smaragdova & Witt 1936). Dashed line represents one-to-one line, as a goodness-of-fit measure E_2_ is shown, which quantifies the difference of the points to the one-to-one line. Dashed line represents one-to-one line, as a goodness-of-fit measure E_2_ is shown, which quantifies the difference of the points to the one-to-one line.

Fig. I6: **Fit of self-predictions using EDM**. Left panel shows *Aleuroglyphus agilis* (prey) and the right panel shows *Cheyletus eruditus* (predator) (Gause, Smaragdova & Witt 1936). Dashed line represents one-to-one line, as a goodness-of-fit measure E_2_ is shown, which quantifies the difference of the points to the one-to-one line.

**Appendix II**: R Code for calculating **Fractions of prediction error (rmse)**

Fractions rsme

knitr::opts_knit$set(root.dir = '~/code/rEDM/200825')

## Example 1: PS-system prey time series

source("interpolate_parameciumdata_200825.R")

chaos_amount_pa_pred <- matrix(nrow=19, ncol = 3)

colnames(chaos_amount_pa_pred) <- c("NonLinearity", "Chaos", "Stochasticity")
for (i in 1:19){

N <- int.data[[i]][2,]

sout = rEDM::s_map(N,E=2, silent =T)

#nonlinearity
theta_var_best = min(unlist(sout$rmse))^2
theta_var_0 =(sout$rmse[[1]])^2
best_theta = sout$theta[which.min(sout$rmse)]
var_part_theta = theta_var_0-theta_var_best

#chaos
sout_t1 = rEDM::s_map(N,E=2,theta = best_theta,tp = 1, silent = T)
sout_t2 = rEDM::s_map(N,E=2,theta = best_theta,tp = 2, silent = T)

l_est <- log(sout_t2$rmse/sout_t1$rmse)

#partitioning
var_total <- theta_var_0
if(l_est > 0) {
 var_part_chaos_fraction <- (theta_var_best-theta_var_best/exp(l_est))/var_total
} else {
var_part_chaos_fraction <- 0}
chaos_amount_pa_pred[i, "NonLinearity"] <- (var_part_theta_fracion = var_part_theta/var_total)
chaos_amount_pa_pred[i, "Chaos"] <- var_part_chaos_fraction
chaos_amount_pa_pred[i, "Stochasticity"] <-
 (var_part_soch_fraction = 1-var_part_theta_fracion-var_part_chaos_fraction)
}

## Resulting mean of experimental replicates

c(mean(chaos_amount_pa_pred[,1], na.rm =T),
mean(chaos_amount_pa_pred[,2], na.rm =T),
mean(chaos_amount_pa_pred[,3], na.rm =T))

## [1] 0.3909581 0.2304617 0.3785803

## Example 2: Chaotic model (May, 1976)

f = function(N0 = 0.1, r = 3, tmax = 15, sigma = 0.2) {
 Nlst = numeric(tmax)
 Nlst[1] = N0
 for(i in 2:tmax) {
 Nlst[i] = Nlst[i-1]*exp(r*(1-Nlst[i-1]))
 }

 rnorm(length(Nlst), Nlst, sigma)
}

#simulate data
set.seed(123)
N = f(r= 3, tmax = 15, sigma = 0.2)
plot(N, type = "l")


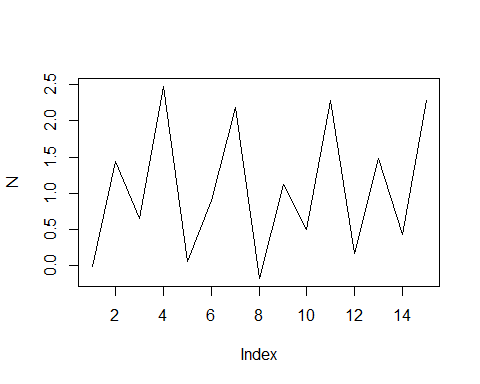


sout = rEDM::s_map(N,E=2, silent =T)

#nonlinearity
theta_var_best = min(unlist(sout$rmse))^2
theta_var_0 =(sout$rmse[[1]])^2
best_theta = sout$theta[which.min(sout$rmse)]
var_part_theta = theta_var_0-theta_var_best

#chaos
sout_t1 = rEDM::s_map(N,E=2,theta = best_theta,tp = 1, silent = T)
sout_t2 = rEDM::s_map(N,E=2,theta = best_theta,tp = 2, silent = T)
l_est <- log(sout_t2$rmse/sout_t1$rmse)

#partitioning
var_total <- theta_var_0
if(l_est > 0) {
 var_part_chaos_fraction <- (theta_var_best-theta_var_best/exp(l_est))/var_total
} else {
 var_part_chaos_fraction <- 0}
nonlinear_may <- (var_part_theta_fracion = var_part_theta/var_total)
chaos_may <- var_part_chaos_fraction
stoch_may <- (var_part_soch_fraction = 1-var_part_theta_fracion-var_part_chaos_fraction)

## Resulting fractions

c(nonlinear_may, chaos_may, stoch_may)

## [1] 0.5250983 0.2199410 0.2549607

Tab. II1: **Fractions of prediction error (rmse)**

|  | PS prey | PS pred | AC prey | AC pred | May (sigma=0.2; r =3) | May  (sigma = 0.5; r =2) | May (sigma =0, r=3) |
| --- | --- | --- | --- | --- | --- | --- | --- |
| Nonlinearity | 0.423 | 0.391 | 0.777 | 0.653 | 0.347 | 0.004 | 0.892 |
| Chaos | 0.273 | 0.230 | 0.132 | 0.161 | 0.315 | 0.006 | 0.002 |
| Stochasticity | 0.304 | 0.379 | 0.091 | 0.187 | 0.338 | 0.99 | 0.106 |
| total rsme | 0.537 | 0.19 | 177.948 | 10.406 | 0.638 | 0.417 | 0.503 |
